# The Influence of Coping and Personality Styles on Satisfaction with Life in Patients with Chronic Kidney Disease

**DOI:** 10.5334/pb.518

**Published:** 2020-03-03

**Authors:** José Manuel García Montes, María José Sánchez Elena, Matías Valverde Romera

**Affiliations:** 1 University of Almería, ES

**Keywords:** Chronic Kidney Disease, Satisfaction with life, Personality Styles, Coping Strategies

## Abstract

The objective of this *ex post facto study* was to find out how different coping and personality styles influence satisfaction with life in a group of 55 people with chronic kidney disease, 34 of whom were receiving haemodialysis and 21 had undergone a kidney transplant. The participants completed three questionnaires, the SWLS, CAEPO and MIPS. The results showed the relationship between active coping strategies and satisfaction with life in haemodialysis patients, kidney transplant recipients and the total sample. A Pleasure-Enhancing personality style was significantly related to Satisfaction with Life, both in the total sample, and in the two groups separately. There were no significant differences in Satisfaction with Life between the haemodialysis patients and kidney transplant recipients. The theoretical repercussions of these results are discussed, highlighting their applications to clinical practice, in which training in active coping is essential.

## Introduction

Chronic Renal Disease (CRD) is a gradual loss of kidney function due to processes which have extensively and permanently damaged both kidneys. In the first stages of the illness, renal function may be normal, as expressed by the Glomerular Filtration Rate (GFR) > 90 ml/min/1.73 m^2^. However, CRD evolves in a severe decline in GFR to later renal failure (GFR) < 15 ml/min/1.73 m^2^, leading to a need for kidney replacement (NKF, 2002).

In Spain, as in many of its neighbouring countries, haemodialysis is the most commonly used treatment for CRD (SEN, 2009). Treatment regimens and their side effects may lead to biopsychosocial problems, among which are sleep disorders, cramps, dermatitis, financial difficulties, dependency, problems at work and with free time (Ekelund & Andersson, 2007). In the literature, results continue to point towards high levels of anxiety and depression among the emotional problems of haemodialysis patients. Páez, Jofré, Azpiroz, and De Bortoli (2009), for example, noted depression in 56.7% of the participants in their study.

Kidney transplantation is the most desirable option in kidney replacement treatment, there is wide consensus that kidney transplantation offers the best quality of life in most cases, even equivalent to that of the general public (Valdés & Ortega, 2006). However, as few as 25%–30% of haemodialysis patients are eligible for receiving a transplant (De Francisco & Otero 2003). Due to advances in surgery and immunosuppressive treatments, the percentage of survivors in the first year after receiving a transplant in Spain today is over 95% (López & Del Castillo, 2007). Immunosuppressive treatment is administered to the patient during surgery. This treatment avoids immune system rejection of the transplanted organ, but also involves serious side effects. The most frequent psychological disorders found among kidney transplant patients are sexual disorders, depression, anxiety, fantasies about the donor, and feelings of dissatisfaction with their physical appearance (Pérez, Martín, & Galán, 2005).

Organ donations in Spain are the highest of all European countries, maintaining a level comparable to that of the United States (ONT, 2010). In the last 10 years, the number of cases of CRD in Spain seems to have stabilised at about 130 per million population (Luño, 2007; SEN 2009).

Whether the CRD treatment is haemodialysis or transplantation, due to the limitations imposed by it, the patient continues suffering from chronic illness and/or disability, conditions that research has related negatively to life satisfaction (Gaines, Marx, Ballew, & Parrish, 2008; Strine, Chapman, Balluz, Moriarty, & Mokdad, 2008). However, other studies, such as those by Pagán-Rodríguez (2010) with longitudinal data provided by the German Socio-Economic Panel (GSOEP), found that, although in the beginning, disability of men of working age has a significant negative effect on satisfaction with life, there is a period of adaptation, after which satisfaction levels are similar to people with no disability. The time lapse, along with the reversibility or chronic condition of the illness, produces some interesting, although paradoxical, reactions to adaptation. Along the same lines, Smith, Loewenstein, Jankovich, and Ubel (2009) found that people who are aware that their health condition (e.g., colostomy) is irreversible are more satisfied with life than those who only live with the condition temporarily.

In the case of haemodialysis patients, Kimmel et al. (1995) found, among other results, that general satisfaction with life had no relationship to the Karnofsky Index, which objectively measures the level of functional performance for activities in daily living. An interesting question might therefore be which psychological factors do actually make a difference in the level of satisfaction with life of CRD patients.

One possible answer may lie in the concept of ‘coping’. In effect, since Lazarus & Folkman defined coping as “constantly changing cognitive and behavioral efforts to manage specific external and/or internal demands that are appraised as taxing or exceeding the resources of the person” (Lazarus & Folkman, 1984, p. 164), its role in psychological adjustment to chronic illness and disability has been widely studied (e.g., Livneh & Parker, 2005; De Ridder, Geenen, Kuijer, & Van Middendorp, 2008). The latest reviews in the field of chronic illness insist that success in psychological adjustment depends on the ability of those affected to “remain as active as possible, recognise and express their emotions in such a way as to permit them to control their lives, to engage in self-care, and concentrate on being positive towards the results of their illness” (De Ridder et al., 2008, p. 246). During this process, the social role of the family is important for coping to be effective (Díaz & Yaringaño, 2010).

For CRD patients undergoing haemodialysis, successful control of coping in such a stressful situation, which requires long-term changes in behaviour, is essential (Quinan, 2007). Self-control can be encouraged by providing patients with information, and is manifested in better adhesion to treatment and self-care. Studies in haemodialysis patients highlight the benefits of an internal locus of control providing better adjustment to illness (Cvengros, Christensen, & Lawton 2005). Furthermore, the role of social support from the dialysis staff has also been confirmed as an effective tool in improving adhesion to the restriction of liquids in haemodialysis patients (Yokoyama et al., 2009).

All of the abovementioned findings contribute to effective coping with chronic illness, psychological intervention, and support from family members and healthcare staff.

However, for people who must cope with chronic illness, satisfaction with life could depend on more stable variables, such as personality styles. A review of the literature found no studies relating life satisfaction to personality styles in patients with chronic illness or disability, in spite of the fact that personality is a factor which is classically related to health psychology. Authors, such as Castro Solano, Casullo, & Pérez (2004), have applied the Millon Index of Personality Styles (MIPS, Millon, 2001), derived from the ecological-evolutionist model, to people with physical illnesses (dermatological, cardiovascular, gastrointestinal, respiratory, glaucoma) to find out whether personality styles in patients with physical illnesses differed from the general population, with affirmative results. The authors used six MIPS scales, Pleasure-Enhancing, Internally Focused, Sensing, Feeling-Guided, Withdrawing and Submissive, which explained 36% of the variance, and participants with a physical illness scored higher than the general population. Moncada et al. (2009) also applied the MIPS in a study on bruxism, and found significant differences between groups with and without that disorder. In that study, subjects without bruxism showed a more adaptive personality profile. They were more optimistic, more active in modifying their environment, more communicative, and surer of their social environment. On the other hand, bruxism was associated with the Pain-Avoiding, Withdrawing, Submissive and Cooperative Subscales.

The main goal of this study was to find out whether general satisfaction with life of CRD patients has any relationship to coping and personality styles. We posed the following hypotheses:

1. General satisfaction with life correlates positively with active coping strategies, both in a combined sample of haemodialysis patients and kidney transplant recipients, and in the two groups separately.
2. General satisfaction with life correlates positively with personality styles considered to be adaptive, both in the combined sample of haemodialysis patients and kidney transplant recipients, and in the two groups separately.

## Methods

### Participants

Fifty-five CRD patients participated in this study, 34 of whom were on haemodialysis and 21 who had received a kidney transplant, all residents of the Province of Almería, Spain. Out of the 34 patients on haemodialysis, 28 were on kidney transplant waiting lists, and the remaining six were ineligible for a kidney transplant for medical reasons.

Of the 35 men and 20 women participants 21 men and 14 women were in the haemodialysis group, and 14 and 7 in the kidney transplant group, respectively.

Inclusion requirements were age 18 to 65 and a minimum formal education. The mean participant age was 41.54 years (*SD* = 9.73) and the average age at diagnosis was 25.85 years (*SD* = 15.34). They had been receiving treatment, (haemodialysis or after transplantation), for an average of 5.31 years (*SD* = 5.16). Table 1 shows other sample characteristics.

**Table 1:** General characteristics of the sample (n = 55).

	Yes	No

Are you married?	37	18
Do you have any children?	28	27
Do you feel supported by your family?	53	2
Have you already received a transplant?	19	36
Do you have from any other illnesses?	24	31
Is there any previous history of CRD in your family?	9	46
Are you still able to work?	23	32
Are you religious?	43	12

### Instruments

An ad hoc questionnaire was used for collecting sociodemographic data, such as age and sex, current treatment and time in their present situation, and other information.

*The Satisfaction with Life Scale* (*SWLS*, Diener, Emmons, Larsen, & Griffing, 1985). The SWLS gives a single score evaluating the participants’ overall opinion of their satisfaction with life. The reliability of the Spanish version used in this study is high, as found by a Cronbach’s Alpha of 0.82, indicating adequate internal consistency (Cabañero, Richart, Cabrero, Orts, Reig, & Tosal, 2004).

*Cuestionario de Afrontamiento al Estrés en Pacientes Oncológicos* [Stress Coping Questionnaire for Cancer Patients] (*CAEPO*, González 2004). The CAEPO consists of 40 multiple choice items ranging from 0 (never) to 3 (almost always), and measures coping strategies and general coping styles of individuals with cancer or other disabling chronic or serious illnesses (González, 2004). The CAEPO yields a transformed score of coping styles of –9 to 9, of which scores of –9 to –3 are possible, and where –1 to –2 shows preferentially negative coping, 0 shows undefined coping, 1 to 2 is preferentially positive coping and 3 to 9 is positive coping. The questionnaire also provides direct scores on seven scales, which differentiate the following most frequently used coping strategies: Confrontation and active struggle (Ela), Self and emotional control (Ace), Search for social support (Bas), Anxiety and overworry (Apa), Passivity and resignation (Prp), Escapism and estrangement (Hd), Denial (Neg). CAEPO reliability has been proven to be high, with an average Cronbach’s alpha of 0.88, indicating internal consistency (González, 2004). The CAEPO reliability construct was found by factor analysis (González, 2004). The saturation weight of each of the seven scales is from 0.64 to 0.87.

The *Millon Index of Personality Styles* (*MIPS*, Millon 2001). This instrument, designed by T. Millon for normal personality styles, applies some of his concepts and contributions to personality disorders. Its theoretical basis includes both historical and theoretical findings (Freud & Jung) as well as other sciences. The MIPS consists of 180 True (T)/False (F) items that gather information in three personality dimensions, Motivating Styles, Thinking Styles, and Behaving Styles, which together group 24 traits, characteristics, and interpersonal styles. Measurements on the questionnaire’s 24 subscales are comprised of opposite pairs that refer to theoretically contrasting styles. However, in a psychometric sense, they are not opposite, as each pole is measured by independent subscales. The subscales that measure the Motivating Styles are Pleasure-Enhancing, Pain-Avoiding, Actively Modifying, Passively Accommodating, Self-Indulging and Other-Nurturing. The Thinking Style subscales are Externally Focused, Internally Focused, Sensing and Intuiting, Thought-Guided, Feeling-Guided, Conservation-Seeking and Innovation-Seeking. And finally, the subscales used to measure Behaving Styles are Withdrawing and Gregarious Retiring, Hesitating and Asserting, Dissenting and Conforming, Submissive and Dominant, Dissatisfied and Cooperative. The adapted Spanish version of the questionnaire also includes three validity indices to counteract possible bias in answers, Positive Impression, Negative Impression and Consistency. A final MIPS measurement, known as the Adjustment Index, estimates the extent to which the person adapts to his reference group and to his surroundings. Reliability and validity of the Spanish adult version of the MIPS measured with the Cronbach’s alpha is 0.73 for women and 0.71 for men.

### Procedure

Before carrying out this study, permission was received from the management of the six haemodialysis centres, as well as the Transplant Review Board to inform CRD patients about the objectives of the study. Afterwards, those who were interested in participating were informed individually that the study would be voluntary and anonymous and were offered the possibility of completing the questionnaires alone or with an interviewer, and 91% of the participants preferred to complete it with the interviewer.

The participants received no economic or other compensation.

## Results

Table 2 shows all the scores and standard deviations on the instruments applied.

**Table 2:** Descriptive Statistics of the combined sample (N = 55), haemodyalisis group (N = 34) and transplant group (N = 21).

Instruments		TOTAL SAMPLE		HAEMOD. GROUP		TRASPLANT. GROUP	

		Average	D.T.	Average	D.T.	Average	D.T

SWLS	Satisfaction	14.31	5.50	13.79	5.80	15.14	4.99
	CAEPO style	0.98	2.87	0.67	2.92	1.47	2.78
	Ela	21.24	4.67	21.17	5.18	21.33	3.82
	Ace	14.80	3.76	14.85	4.01	14.71	3.39
CAEPO	Bas	6.76	3.18	6.26	3.15	7.57	3.13
	Prp	9.51	3.52	10.20	3.30	8.38	3.65
	Hd	7.25	3.10	7.61	3.05	6.66	3.16
	Apa	5.89	3.64	5.32	3.48	6.80	3.77
	Neg	2.52	1.79	2.58	2.00	2.42	1.43
	Pleasure-Enhancing	64.62	22.56	66.79	22.43	61.09	22.86
	Pain-Avoiding	41.07	22.99	39.20	21.56	44.09	25.37
	Actively Modifying	53.69	22.47	52.97	22.94	54.85	22.19
	Passively Accomod	52.47	24.29	55.35	23.71	47.80	25.07
	Self-Indulging	45.34	23.90	48.76	23.79	39.80	23.58
	Other-Nurturing	64.73	24.06	62.32	23.99	68.61	24.22
	Externally Focused	58.85	24.99	58.29	24.49	59.76	26.36
	Internally Focused	45.14	21.45	41.88	20.98	50.42	21.64
	Sensing	63.04	22.98	62.76	23.57	63.47	22.56
	Intuiting	43.54	22.13	41.73	19.75	46.47	25.75
	Thought-Guided	47.09	23.19	47.26	25.01	46.80	20.48
	Feeling-Guided	61.31	24.64	59.70	24.41	63.90	25.39
	Conservation-Seek	54.47	22.49	51.44	21.62	59.38	23.52
MIPS	Innovation-Seeking	43.69	25.75	44.50	24.44	42.38	28.32
	Withdrawing	46.76	24.95	46.11	25.24	47.80	25.05
	Gregarious	54.02	24.79	55.61	24.39	51.42	25.80
	Hesitating	43.71	25.42	42.70	24.78	45.33	26.94
	Asserting	52.67	24.77	54.82	25.00	49.19	24.59
	Dissenting	43.56	27.76	42.17	26.78	45.80	29.82
	Conforming	61.42	23.33	60.73	21.92	62.52	25.95
	Submissive	47.09	23.81	42.61	21.28	54.33	26.34
	Dominant	44.71	24.05	46.97	23.78	41.04	24.61
	Dissatisfied	43.20	28.16	41.50	29.34	45.95	26.59
	Cooperative	71.40	22.19	71.23	21.44	71.66	23.88
	Positive Impression	4.07	1.99	4.29	1.89	3.71	2.12
	Negative Impression	3.96	2.53	3.91	2.37	4.04	2.81
	Consistency	3.62	1.11	3.52	1.02	3.76	1.26
	Adjustment Index	46.09	14.50	47.85	13.88	43.23	15.37

The Pearson Correlation Coefficient was employed to determine the relationships between the study variables. Table 3 shows the significant correlations between the Satisfaction with Life variable and the coping measurements in the three groups studied.

**Table 3:** Correlation between the satisfaction with life and coping variables.

Satisfaction with life	CAEPO Style	Ela	Ace	Bas	Prp	Hd	Apa	Neg

COMBINED SAMPLE	0.548**	0.494**	0.520**	0.310*	–0.121	–0.156	–0.436**	0.259
HAEMODYALISIS GROUP	0.509**	0.565**	0.512**	0.264	0.129	–0.252	–0.516**	0.279
TRANSPLANT GROUP	0.606**	0.319	0.560**	0.352	–0.483*	0.063	–0.404	0.235

* p < 0.05; ** p < 0.01.

The significant correlations found between the Satisfaction with Life variable and the personality measurements are shown in Table 4.

**Table 4:** Correlation between the satisfaction with life and personality variables.

Satisfaction with life	COMBINED SAMPLE	HAEMOD. GROUP	TRANSPLANT GROUP

Pleasure-Enhancing	0.533**	0.589**	0.498*
Pain-Avoiding	–0.492**	–0.549**	–0.466*
Actively Modifying	0.267*	0.287	0.219
Passively Accommodating	–0.398**	–0.433*	–0.283
Externally Focused	0.467**	0.410*	0.576**
Internally Focused	–0.422**	–0.433*	–0.508*
Conservation-Seeking	0.294*	0.298	0.252
Withdrawing	–0.476**	–0.514**	–0.427
Hesitating	–0.500**	–0.545**	–0.459*
Asserting	0.311*	0.369*	0.252
Dissenting	–0.392**	–0.409*	–0.402
Conforming	0.272*	0.190	0.409
Submissive	–0.321*	–0.425*	–0.283
Dissatisfied	–0.509**	–0.570**	–0.425
Adjustment Index	0.527**	0.631**	0.439*
Negative Impression	–0.607**	–0.670**	–0.544*

* p < 0.05; ** p < 0.01.

A linear equation model was used to check the extent to which the coping and personality scales predicted Satisfaction with Life in the combined sample of haemodialysis patients and kidney transplant recipients. Variables predicting the CAEPO and MIPS subscales were entered as predictive variables for participant scores on the SWLS. Stepwise regression was used with a probability of F for inclusion of <.05 and of F > .100 for exclusion. Thus, the final equation was significant (F (2.52) = 17.080, *p* = .000) for the CAEPO style and enhancement variables which explained more than 37.3% of the variance (adjusted R^2^ = .373). Table 5 summarizes the results of these analyses.

**Table 5:** Multiple regression analyses of the combined sample.

Step	Dependent variable	Variables entered	R^2^	Adjusted R^2^	Std. error of the estimate	Durbin-Watson Statistic	β	t	p	Collinearity statistics	

										Tolerance	VIF

**Step 1**	Satisfaction	CAEPO Style	.300	.287	4.647		.548	4.766	.000	1.000	1.000
**Step 2**	Satisfaction	CAEPO Style	.396	.373	4.357	1.688	.381	3.116	.003	.776	1.288
		Expansion					.353	2.883	.006	.776	1.288

The same statistical procedure applied to the dialysis group yielded a significant final equation (F (1.32) = 21.124, p = .000). For this group the MIPS Adjustment Index explained 37.9% of the variance (R^2^ corrected = .379). A summary of the results are found in Table 6.

**Table 6:** Multiple regression analyses for the haemodyalisis group.

Step	Dependent variable	Variables entered	R^2^	Adjusted R^2^	Std. error of the estimate	Durbin-Watson Statistic	β	t	p	Collinearity statistics	

										Tolerance	VIF

**Step 1**	Satisfaction	MIPS adjustment Index	.398	.379	4.578	2.209	.631	4.596	.000	1.000	1.000

Finally, the statistical procedure was applied to the kidney transplant group, resulting in a significant final equation (F (1,19) = 11.02, p = .004). For this group, the CAEPO style explained 33.4% of the variant (R^2^ corrected = .334). The results for this group are shown in Table 7.

**Table 7:** Multiple regression analyses for the transplant group.

Step	Dependent variable	Variables entered	R^2^	Adjusted R^2^	Std. error of the estimate	Durbin-Watson Statistic	β	t	p	Collinearity statistics	

										Tolerance	VIF

**Step 1**	Satisfaction	CAEPO Style	.367	.334	4.075	2.42	.606	3.320	.004	1.000	1.000

The test for inter-subject factors showed that the results for the haemodialysis and kidney transplant groups were similar, as there were no significant differences between one group and the other. Nor were differences found for Satisfaction [F (1, 53) = .77, p = .38], CAEPO style [F (1, 53) = 1.00, p = .32], Ela [F (1, 53) = .01, p = .90], Ace [F (1, 53) = .01, p = .89], Bas [F (1, 53) = 2.23, p = .14], Prp [F (1, 53) = 3.65, p = .06], Hd [F (1, 53) = 1.22, p = .27], Apa [F (1, 53) = 2.21, p = .14], Neg [F (1, 53) = .10, p = .75], Pleasure-Enhancing [F (1, 53) = .82, p = .36], Pain-Avoiding [F (1, 53) = .58, p = .44], Actively Modifying [F (1, 53) = .09, p = .76], Passively Accommodation [F (1, 53) = 1.25, p = .26], Self-Indulging [F (1, 53) = 1.85, p = .17], Other-Nurturing [F (1, 53) = .88, p = .35], Externally Focused [F (1, 53) = .04, p = .83], Internally Focused [F (1, 53) = .2.10, p = .15], Sensing [F (1, 53) = .01, p = .91], Intuiting [F (1, 53) = .59, p = .44], Thought-Guided [F (1, 53) = .00, p = .94], Feeling-Guided [F (1, 53) = .37, p = .54], Conservation-Seeking [F (1, 53) = 1.63, p = .20], Innovation-Seeking [F (1, 53) = .08, p = .77], Withdrawing [F (1, 53) = .05, p = .81], Gregarious [F (1, 53) = .36, p = .54], Hesitating [F(1, 53) = .13, p = .71], Asserting [F (1, 53) = .66, p = .41], Dissenting [F (1, 53) = .21, p = .64], Conforming [F (1, 53) = .07, p = .78], Submissive [F (1, 53) = 3.27, p = .07], Dominant [F (1, 53) = .78, p = .38], Dissatisfied [F (1, 53) = .32, p = .57], Cooperative [F (1, 53) = .00, p = .94], Positive Impression [F (1, 53) = 1.10, p = .29], Negative Impression [F (1, 53) = .03, p = .84], or Consistency [F (1, 53) = .56, p = .45].

## Discussion

As proposed in Hypothesis 1, general Satisfaction with Life correlated positively with active coping strategies, both in the combined sample and in the haemodialysis and kidney transplant groups separately. In the combined sample, it was found that the three most adaptive strategies for coping, Ela, Ace and Bas, were positively related to Satisfaction with life. In the haemodialysis group two of these variables (Ela and Ace) were related to it, and in the kidney transplant group, only one (Ace) was related. However, although no relationship between Satisfaction with Life and any of the possible active coping strategies appeared, the relationship found in the haemodialysis and kidney transplant groups was in the direction proposed for Hypothesis 1.

According to Hypothesis 2, general Satisfaction with life correlates positively with adaptive personality styles, both in the combined sample, and in the haemodialysis and kidney transplant groups separately. This second hypothesis can be understood as partly confirmed.

Positive relationships were also found in the combined sample between Satisfaction and the Pleasure-Enhancing, Externally Focused, Confident, Conservation-Seeking, Conforming, Actively Modifying variables and the Adjustment Index. The negative correlations referring to the less adaptive scales, Dissatisfied, Hesitating, Withdrawing, Internally Focused, Dissenting, Passively Accommodating and Submissive, support these results. The motivational, cognitive and interpersonal personality areas covered by the MIPS are also observed in the scales mentioned above.

In the haemodialysis group, the results demonstrated that there was a direct relationship between Satisfaction and the Adjustment Index, Pleasure-Enhancing, Externally Focused and Asserting. The opposite relationship between Satisfaction and the Dissatisfied, Pain-Avoiding, Hesitating, Withdrawing, Internally Focused, Passively Accommodating, Submissive and Dissenting scales also supported this second hypothesis.

In the kidney transplant group, a direct relationship was found between Satisfaction with Life and the Externally Focused, Pleasure-Enhancing scales and with the Adjustment Index, and the opposite was found with Internally Focused, Pain-Avoiding and Hesitating. As for the first hypothesis, fewer correlations were found in this group than in the haemodialysis group and the combined sample. These results may be due to the fact that the kidney transplant group had fewer participants. Even so, the data are congruent with the proposed hypothesis.

The Pleasure-Enhancing variable was the most outstanding due to its involvement in the equations that predict greater Satisfaction with Life, as well as its positive relationship with this variable in all three groups. It is interesting that the Satisfaction and Adjustment Index variables correlated in the same direction and practically with the same variables. This result could indicate, among others, that these two variables are similar, meaning that they measure similar constructs.

As mentioned above, no statistically significant differences were found between the haemodialysis and kidney transplant groups. Although it is accepted that quality of life is poorer for haemodialysis patients than kidney transplant recipients (Valdés & Ortega, 2006), the lack of a significant difference in life satisfaction is coherent with other studies, demonstrating that this construct is relatively independent of the objective situation or physical limitations the person suffers from, as also found by Kimmel et al., (1995). These data are also be consistent with the findings of Pagán-Rodríguez (2010) in disabled men of working age, in the sense that even though they were suffering from a chronic illness or disability, adaptation to their situation over time placed the satisfaction felt by such people on the same level as the general population.

In social relationships, as for people with other chronic illnesses (Gaines et al., 2008; Strine et al., 2008), social support is an important factor related to life satisfaction for dialysis patients (Kimmel et al., 1995). Social support has also been proven to help haemodialysis (e.g., Yokoyama et al., 2009) and cancer patients (e.g., Díaz & Yaringaño, 2010) feel able to cope positively. In this study, social support was not studied directly, although some of the results were coherent with relevant literature. One such case is the positive relationship between coping strategy, Bas (search for social support), and Satisfaction in the combined sample. The MIPS also provided important measurements that, once more, relate a higher level of Satisfaction with Life to people who have a more social predisposition, as seen in the relationship between Satisfaction with Life and Asserting, whereas the opposite applies to Withdrawing and Hesitating.

The results found in this study coincide with the factors facilitating positive coping in chronic illnesses reported by de Ridder et al., (2008). Thus, the overall positive relationship of our sample between life satisfaction and active coping strategies, particularly the Ela (coping and active struggle) and Ace (emotional self-control) scales, supports the recommendation to “be as active as possible, to recognise and express emotions in such a way that this allows them to take control of their lives, to take an active part in their self-care and to concentrate on the results of their illness being positive” (p. 246). Our results are also in agreement with the premises of González (2004) and the results described by Díaz & Yaringaño (2010) on positive coping by cancer patients, as expressed by the relationship between Satisfaction with Life and the Ela, Ace and Bas coping factors.

Other studies which have used the MIPS in subjects with chronic illnesses have shown similar results. For instance, Castro Solano, Casullo, and Pérez (2004), using six MIPS scales (Pleasure-Enhancing, Internally Focused, Feeling Guided, Sensing, Feeling-Guided, Withdrawing and Submissive) were able to explain 36% of the variance of a sample of a population with physical disabilities. Three of these scales (Pleasure-Enhancing, Sensing and Feeling-Guided) showed high means in our study. However, in a study by Moncada et al. (2009) on variables associated with bruxism, Pain-Avoiding, Withdrawing, Submissive and Cooperative, only Cooperative is in agreement with our study, insomuch as it showed the highest mean of all the personality scales.

Possible clinical applications of these results include training haemodialysis and renal transplant patients in the most adaptive coping strategies. Both treatments show similarities in that they require following a strict self-care routine, although this is generally more limiting for haemodialysis patients. At the same time, this attitude may seem contradictory for them, considering that these patients must also remain in a passive position when receiving their treatment. Overall, they must accept that the end of haemodialysis depends more on outside factors like the appearance of a compatible donor than their personal behaviour. In this situation, these patients’ lives lack motivation and they fail to follow medical prescriptions, leading to worsening health.

Among the limitations of this study, the small sample size and the inability to draw causal inferences about the relationship between variables should be clearly stated.

Although it was found that some dependent variables were able to predict life satisfaction of the participants, it should be borne in mind that the study design was merely correlational and these results are based only on correlations found between the variables. Longitudinal studies are necessary if it is desired to establish causal relationships, and this is proposed as an objective for future research. When evaluating the limitations of the study, it should also be considered that the conditions under which the questionnaires were completed, as mentioned in the procedure, may have caused variations in the answers given by the participants.

According to our results, and in view of the limitations mentioned above, the study of patients with chronic illnesses and their life satisfaction provides an opportunity for comparison and progress in understanding their psychological adaptation.
